# Mr. Hunter, on the Influenza

**Published:** 1803-09-01

**Authors:** Alex. Hunter

**Affiliations:** Dumbarton


### *To the Editors of the Medical and Physical Journal.*

GENTLEMEN,

If you think the following remarks on Influenza worthy a place in your Journal, they are at your service.

The disease made its first appearance here about the beginning of April, and spread with astonishing rapidity; the three last weeks of March had been rainy and chill, the wind wind generally west or south west; the mean height of the barometer nearly 29,5, the thermometer about 42, but frequently, during the night, it fell as low as 30; the quantity of rain during that period 1,127 inches.

Its symptoms, in general, were shiverings, severe pain in the head, nausea, frequently vomiting, hard dry cough, compression of the breast, and a sense of soreness about the throat and fauces, as if the skin was off; the breathing was difficult, pulse quick and full at the beginning; and in every case that I saw, the debility was much greater than could have been expected, either from the duration of the disease, or from the severity of the symptoms; no running from the nose or eyes took place in any of my patients, but a number of them were affected in the latter stages of the disease, and some even in the beginning with severe griping pains in the belly, tenesmus, and considerable discharge of slimy matter tinged with blood; in some instances, affection of the joints resembling acute rheumatism took place, which went on from joint to joint, till it affected in its course almost every joint; those were all young people, and the recovery was very tedious.

When the disease was protracted beyond the tenth day, the pulse became gradually weaker and quicker, and brought on a typhus state.

My mode of practice, at the commencement of the disease, was to give a solution of antimon. tart. in small quantity, repeated till it produced vomiting; in this way it frequently caused a few loose stools; after vomiting, the feet were bathed, and perspiration promoted by inhaling the steams of warm water and vinegar; if the antimonial had not procured passage of the belly, laxatives were given; and when stitches and difficulty of breathing were troublesome, blisters were freely used; if the cough was very distressing, sometimes recourse was had to opium. I had pursued this plan for some time, when accident brought me acquainted with a remedy which I think was productive of very beneficial effects.

A young man who had been affected with influenza for twelve days, had been advised by some acquaintance to drink a considerable quantity of whisky; this had exasperated all his symptoms, and brought on so severe a vomiting, that every thing he took was instantaneously rejected; his belly was bound, skin dry, and his pulse 130, weak and intermitting. Before I saw him, a number of injections had been given, some of them very stimulant, but without effect; I ordered an injection of nicotiana ʒij.

infus.

infus, in aq. bullient. ℥xij. to be given; it produced an immediate evacuation of a considerable quantity of indurated fæces, caused severe vertigo, faintishness, and at last a copious perspiration, followed by sound and refreshing sleep; when he awoke his fever was greatly diminished, and the vomiting did not again come on; and without any other application, except a plentiful use of wine, in which a small quantity of rad. gentian was infused, he quickly recovered.

As this remedy had been so useful in this case, I determined to try it in others: and as in the beginning of the disease costiveness was frequent, I began by giving the injection of a strength in proportion to the state of the patient, but always wished, that besides emptying the bowels, nausea, or even vomiting might be excited; whenever this took place, it was followed by a profuse sweat and sound sleep.

After having found the beneficial effects of the medicine in injections, its internal use by the mouth was tried; the preparation made use of was the vinum nicotianæ, given at first in small doses, frequently repeated, combined with some aromatic water or tincture; it was always pushed so far as to produce giddiness of the head, or nausea, and when carried this length, it almost in every instance brought on a large discharge by the skin and kidneys, and sound sleep; it also caused a considerable increase of expectoration, and relieved the cough; indeed, in my opinion, its anodyne effects were equal to those of opium; and as it aided the discharge from the breast, which the other retarded, it was undoubtedly preferable.

Since I first became acquainted with the sudorific and narcotic powers of the nicotiana, it has been used in every case that came under my management, except those where gripes and bloody, slimy stools took place, and always with evident advantage; nor did I see, in any one instance, the least disagreeable circumstance arise from its use.

When, after the first exhibition of the medicine, any fever remained, and the cough was troublesome, a second dose was given in the course of two or three days after the first, and managed just in the same way; the operation was equally successful as before, and the symptoms proportionally diminished; a third application I never had occasion to make.

I wish however to remark, that during the course of a month that the medicines, as mentioned in the beginning of this paper, were used, my practice was tolerably successful, but not equal to what it was afterwards, as my patients tients both recovered with less pain, and in a much shorter period, by the use of nicotiana, than by all the other remedies formerly employed.

At the same time, however powerful this medicine might appear to be, when stitches existed, blisters were always employed, and frequently the use of steam, by way of fomentation, to the fauces and bronchia.

In those cases which affected the joints, an external application of ol. terebinthæ et sp. cornu cervi, p. æ. was applied every night, and the parts afterwards covered with flannel, but the utility of the external application was much aided by the sudorific effects of the nicotiana.

When diarrhœa, with gripes and slimy stools tinged with blood, took place, pulv. jallapæ comp. was used, solutions of the neutral purging salts, and tinct. theb. in proportion to the pain; after free evacuation, cordials and stomachic bitters with wine.

I may probably have thought more of the medicine mentioned above than it deserves, it may not be so successful in another situation as here; but from all I have observed of its mode of action, no ill effects can happen from giving it a trial; even in cases of catarrh, where copious perspiration is required, it will probably have considerable success.

In two cases that occurred here, the influenza was undoubtedly produced by animal infection; one of the persons was a farmer, who went to sow corn for a neighbour that had the disease; to hold the seed they gave him a sheet upon which a sick man had lain, and perspired during the night; it was scarcely tied round his neck, when he found the effluvia produce squeamishness; he was seized with shiverings before he left the field, and his symptoms were nearly the same as those of the person from whom he received the infection.

The second was a young man in perfect health, who came to visit his brother labouring under influenza; he lay that night on a bed which his brother had recently quitted; he found the smell so disagreeable that he could not sleep; in twenty-four hours he was seized with the disease, and his symptoms were nearly similar to those of his brother.

In Vol. IX. No. 48, Page 173, you inserted a case of mine, where a very large quantity of cerussa acetata had been taken without producing any ill effects; since that time I have used it in a few cases of hæmorrhage, both external and internal, and have given it to the extent of two drachms a day without any inconvenience; but although it it may be given with safety, yet in every trial its effects were far inferior to the solutions of sal. mart. of alum, &c. or even to dilute vitrolic acid. I intend trying the subject still further, in order to establish the comparative merits of the acetata.

I am, &c.

*Dumbarton, July* 11, 1803. ALEX. HUNTER.

